# Report on the Treatment of Burns, &c.

**Published:** 1842-01

**Authors:** 


*Report on the Treatment of Burns in the Hospital of La Charite.* Translated and abridged, for the Medical Gazette, from L'Experience, of 30th Sept., 1841.

Burns, admitted into the Hospital of La Charite, in the wards of M. Velpeau, are generally arranged, according to their depth, after the classification of Dupuytren, which comprises six varieties, namely, burns of the first degree, or those attended with an erythematous blush of the skin; burns of the second degree, or those in which the cuticle is raised in blisters; burns of the third degree, or those in which the true skin is involved; burns of the fourth degree, which reach the subcutaneous tissue; burns of the fifth degree, in which the soft parts are deeply involved, and burns of the sixth degree, in which the whole part is destroyed.

M. Velpeau resorts to three principal methods of treating these several varieties. If the burn is of the first degree, and not extensive, simple refrigerants, or the local discutients usually employed, will be sufficient. But Baynton's method of dressing the parts with straps of diachylon plaster, will almost always arrest the evil; the same treatment is applicable to burns of the second and even of the third degree. A single dressing is sufficient for burns of the first degree, and it rarely happens that the cure is not found to be complete, on raising the straps at the end of the third or fourth day. In burns of the second degree, M. Velpeau first raises the epidermis from the blisters, and carefully cleanses the surface beneath, with a linen cloth; he then applies the straps, and usually two or three such dressings will bring about desiccation and cicatrization of the whole surface.

At La Charite, during this year, burns of this variety, which covered almost the whole length of the fore-arm, have been cured in six or seven days, by two dressings only. If the burn is of the third degree, a greater number of dressings will be required, and the cure will rarely be effected in less than fourteen or fifteen days, because the eschars must slough off from the burnt surface before cicatrization can take place.

This very simple method of dressing requires, however, some care; it is not proper, when the affected part is the seat of a decided inflammatory swelling, nor when the burn extends over a large surface. Moreover, it cannot be applied to the face, the neck, the thorax, the abdomen, nor to the extremities, where they are united to the trunk. Hence it is to be used upon the arm, the fore-arm, the hand, the thigh, the leg, and the foot. For other parts, particularly the face, M. Velpeau prefers the liniment composed of equal parts of lime-water and olive-oil, well-beaten together; this produces very happy results, and may be applied four or five times a day, with the feather of a quill. If the burn is on the face, it is not to be covered with dressings, except that it is well to place over it a sheet of scorched paper, after each application of the liniment.

Of the cases which have been treated at La Charite during the summer, the two following may be cited:

A man employed in the privies, set fire to the gas in one of the places which he was cleaning, and the whole of his face, the fore part of his neck, and both his hands, were burnt in the first, second, and third degrees. He was brought into La Charite the day after the accident, with the appearance of having been broiled, and the only application resorted to was the immediate use of the liniment above referred to; the result was, that at the end of nine days after the accident, he was able to go out and attend to his business, his face and arms being entirely free from purulent secretion or excoriation.

*Case* II.—A young girl was severely scalded over the whole face, the forehead, the supra-hyoideal region, and the greater part of both hands, and was admitted into La Charite on the third day, at which time there was erysipelatous swelling and decided reaction over the whole extent of the injured surface. The burn was of the first degree at some points, of the second in others, and of the third in spots over the malar-bones. The parts had been dressed, previous to her admission, with the lead ointment, which was suspended, and the ointment of oil and lime-water was immediately applied, and the pain, heat and swelling began to subside at once; the burnt surfaces diminished by degrees, and on the eighth day the cure was completed.

This is the treatment which the Professor of La Charite recommends as particularly applicable to burns of the three first degrees, when they occupy an extensive surface, or occur in parts difficult to dress, or which are habitually exposed to the air. It is hardly necessary to add, that these two methods of dressing are of little value in burns attended with deep eschars, for in these suppuration and sloughing of the eschars must take place, after which the proper means must be taken to produce cicatrization of the ulcers.

In the treatment of extensive burns of the body and limbs, M. Velpeau has tested the comparative merits of cotton, cold water, and chlorine water, and has come to the conclusion that cotton is not more efficacious than charpie (lint); that cold water produces much the same effects as the chlorine solution: and that simple salt water, so long a popular remedy, is really the best application.

It is necessary to add, that burns which extend over at least half the body, and in which the whole thickness of the skin is involved, rarely fail to destroy the patient during the first two or three days, or when re-action comes on, or else during the process of suppuration, and that in these cases no treatment offers a probable chance of success; but no cases of this kind have occurred among the 18, cited by Mr. Velpeau, in his report of this year.

